# Development of patient-specific phantoms for verification of stereotactic body radiation therapy planning in patients with metallic screw fixation

**DOI:** 10.1038/srep40922

**Published:** 2017-01-19

**Authors:** Dongryul Oh, Chae-Seon Hong, Sang Gyu Ju, Minkyu Kim, Bum Yong Koo, SungBack Choi, Hee Chul Park, Doo Ho Choi, Hongryull Pyo

**Affiliations:** 1 Department of Radiation Oncology, Samsung Medical Center, Samsung Biomedical Research Institute, Sungkyunkwan University School of Medicine, Seoul, 06351, Korea

## Abstract

A new technique for manufacturing a patient-specific dosimetric phantom using three-dimensional printing (PSDP_3DP) was developed, and its geometrical and dosimetric accuracy was analyzed. External body contours and structures of the spine and metallic fixation screws (MFS) were delineated from CT images of a patient with MFS who underwent stereotactic body radiation therapy for spine metastasis. Contours were converted into a STereoLithography file format using in-house program. A hollow, four-section PSDP was designed and manufactured using three types of 3DP to allow filling with a muscle-equivalent liquid and insertion of dosimeters. To evaluate the geometrical accuracy of PSDP_3DP, CT images were obtained and compared with patient CT data for volume, mean density, and Dice similarity coefficient for contours. The dose distribution in the PSDP_3DP was calculated by applying the same beam parameters as for the patient, and the dosimetric characteristics of the PSDP_3DP were compared with the patient plan. The registered CT of the PSDP_3DP was well matched with that of the real patient CT in the axial, coronal, and sagittal planes. The physical accuracy and dosimetric characteristics of PSDP_3DP were comparable to those of a real patient. The ability to manufacture a PSDP representing an extreme patient condition was demonstrated.

Stereotactic body radiation therapy (SBRT), also known as stereotactic ablation radiation therapy, is an RT technique used to deliver very high doses of radiation in a few fractions with a steep dose gradient between the target volume and organ at risk (OAR). Many clinical studies have demonstrated the efficacy and safety of SBRT in the treatment of both benign and malignant diseases^1^. However, small errors during SBRT carry the risk of target under-dose and OAR overdose^2^,^3^,^4^,^5^,^6^,^7^. Thus, proper quality assurance (QA) procedures are imperative for reducing the likelihood of errors in SBRT. Such QA is achieved using pre-treatment patient-specific dose verification, which is a critical component of SBRT meant to ensure that the delivered dose accurately matches the dose distribution determined from the treatment planning system (TPS)^8^,^9^.

Different types of pre-treatment patient-specific dosimetric QA (DQA) techniques have been introduced to confirm patient safety and accurate dose delivery to the target volume for better clinical outcomes^10^. The most common DQA technique is indirect dose verification, which is performed by comparing the calculated and measured doses using a standardized dosimetric phantom (SDP), typically a round-shaped homogeneous solid phantom^11^,^12^,^13^. In making treatment decisions, one of the key factors is evaluating the clinical influence of dosimetric errors detected during the DQA procedure. In addition, accurate dosimetric verification is essential for assessing the dose calculation algorithm in TPS.

However, the results of DQA performed using an SDP have some limitations with respect to determining dosimetric errors in real patients. First, SDPs have a totally different geometric structure compared to patients, including the external body and internal structure. Further, they do not reflect the different densities of structures found in real patients. Thus, SDPs generate different patterns of dose distribution due to different dose absorption and scatter interactions compared with those in real patients. For this reason, accurate prediction of the clinical effects of dosimetric errors is difficult with SDP-based DQA, as the location and degree of dosimetric error differ compared to those of real patients. Second, because the measurement positions and insertion holes are fixed in SDPs, they are not suitable for measuring the dose at the desired region, such as clinically radiosensitive regions like OAR or target volume. Lastly, SDPs have a limited ability to represent extreme patient conditions such as the presence of prosthesis. Indeed, such prosthetic devices usually consist of high-density materials near targets, including hip prostheses, dental prostheses, and spinal fixation devices^14^,^15^,^16^,^17^. The presence of such devices can generate different patterns of absorption and scatter compared to internal organs. Thus, because prosthetic devices are difficult to measure with SDPs, they complicate dose calculation in the TPS, which can result in serious dosimetric errors.

Accurate DQA requires a patient-specific dosimetric phantom (PSDP) that generates the same dose distribution as a real patient, with customizable measurement conditions. Casting or computerized milling machines are typically used to manufacture SDPs. However, these conventional techniques have limited ability to produce PSDPs. We investigated three-dimensional printing (3DP) technology to improve the PSDP manufacturing process^18^. This approach is capable of producing high-resolution structures and is also an excellent design tool that can incorporate the digital imaging and communication in medicine for radiotherapy (DICOMRT) protocol^19^ and computer-aided design (CAD) technology. Some authors have employed 3DP technology to manufacture dosimetric phantoms, but they have exhibited limited usefulness^20^,^21^,^22^,^23^,^24^,^25^.

In the present study, we developed a new technique to manufacture PSDPs using 3DP (PSDP_3DP), which we termed radiation-aided design (RAD). Here, we report the manufacturing procedure for a PSDP_3DP containing metallic fixation screws (MFS) and describe its geometrical and dosimetric accuracy.

## Results

**Figure 1:**
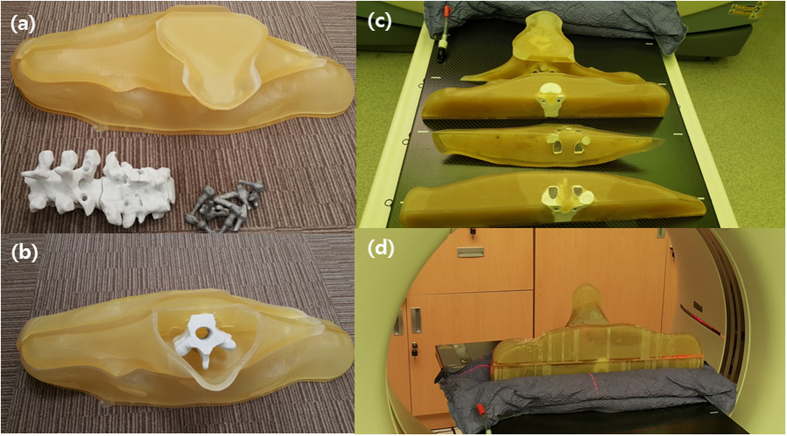
The PSDP_3DP was printed as 20 separate pieces using three different types of 3D printer with compatible materials (**a** and **b**). UV- curable acrylic plastic (UVAP), plastic powder, and titanium were used to create the external body, spine, and metallic fixation screws, respectively, which were then assembled (**c** and **d**).

**Figure 2:**
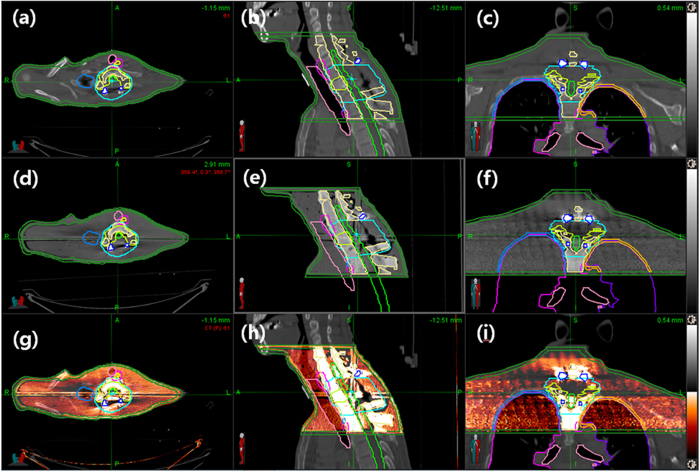
The registered CT of the PSDP_3DP (**d**–**f**) was well matched with that of the real patient CT (**a**–**c**) in the axial, coronal, and sagittal planes (**g**–**i**). The contours of the patient were in good agreement with those of the CT image of the PSDP_3DP (**d**–**f**).

We manufactured the PSDP_3DP using in-house software and three types of 3DP for DQA of a spine SBRT patient without encountering serious issues (Fig. 1). The shape of the PSDP_3DP body was very similar to that of the real patient. Metal artifacts were observed in a similar fashion in both CT image sets (Fig. 2). The registered CT of the PSDP_3DP (Fig. 2d–f) was well matched with that of the real patient CT (Fig. 2a–c) in the axial, coronal, and sagittal planes (Fig. 2g–i). In addition, the contours of the patient were well matched with the CT image of the PSDP_3DP (Fig. 2d–f).

The comparisons of the geometric and dosimetric parameters of the two plans are summarized in Table 1. In comparing the contour volumes between the patient and PSDP_3DP, the percent differences in volume for the external body, spine, and MFS were −4.1% (4438 vs. 4256 cm^3^), 6.4% (156 vs. 166 cm^3^), and 10.0% (20 vs. 22 cm^3^), while the DSCs were 0.98, 0.91, and 0.89, respectively. The percent difference in volume for MFS was slightly higher compared with the other structures, but the absolute volume difference was very small (2 cm^3^).

The mean density of the body shell, the internal body (agar liquid only), and the spine were 1.10 g/cm^3^, 1.05 g/cm^3^, and 1.53 g/cm^3^, respectively. The density of the titanium MFS could not be measured accurately with CT HUs due to limited attenuation of the CT scanner. In comparing densities between the patient and PSDP_3DP, the percent differences for the external body and spine were 7.5% (0.99 ± 0.37 vs. 1.07 ± 0.29 g/cm^3^) and 15.5% (1.29 ± 0.34 vs. 1.53 ± 0.46 g/cm^3^), respectively.

**Figure 3:**
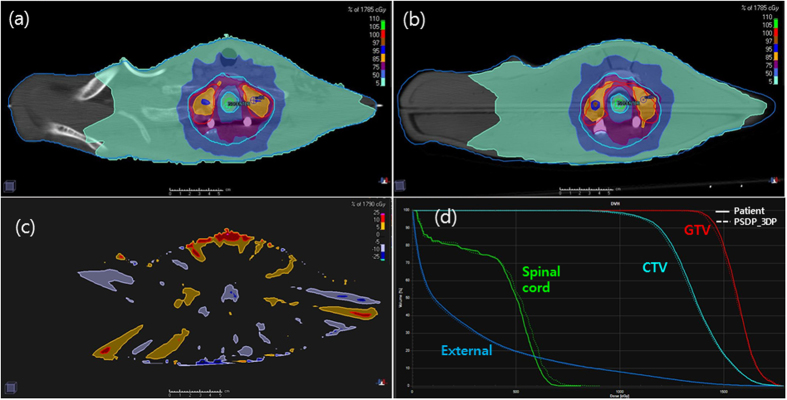
Plans for the patient (**a**) and PSDP_3DP (**b**) showed very similar dose distributions in the axial plane. Larger dose differences were observed at the border of the external body contour (low-dose region), but was minimal in the center region (high-dose region), the difference of which was within 5% according to an axial dose difference map (**c**). The DHVs of the both plans were well matched (**d**).

As viewed from the axial plane at the target center, the two plans based on the real patient and PSDP_3DP showed very similar dose distributions (Fig. 3a and b). The high-dose region was better matched between the patient and PSDS_3DP than the low-dose region, which was depicted on a map of the dose difference between the two plans (Fig. 3c). Large dose differences were observed at the border of the external body contour (low-dose region), while most of the center region (high-dose region) was in good agreement, with a dose difference within 5%.

Compared with the patient plan, the DVH of the PSDP_3DP plan showed a slightly lower dose for GTV, CTV, and external body, while the spinal cord showed a slightly higher dose (Fig. 3d). Generally, however, the DHVs of both plans were well matched. Specifically, the mean differences in dose for GTV, CTV, spinal cord, and external body were −0.5% (1513 vs. 1506 cGy), −0.5% (1311 vs. 1304 cGy), 4.0% (425 vs. 442 cGy), and −2.8% (285 vs. 277 cGy), respectively.

## Discussion

In the present study, a PSDP_3DP of a patient with a prior metallic screw fixation undergoing SBRT of the spine was manufactured using in-house software and three different 3DP technologies. A primary goal of this study was to use the PSDP_3DP to imitate the patient for the purpose of dosimetric evaluation. The PSDP_3DP had acceptable overall geometric and dosimetric accuracy for clinical use; however, the DSCs of the MFS and spine body were slightly lower than that of the external body, which we attributed to misalignment during the assembly process. In addition, contour delineation error due to the influence of the metal artifacts of the MFS was also believed to have contributed to this error. The densities of the external body shell, spine, inner body material (agar liquid) were slightly higher than those of the patient, which led to an increase in overall density of PSDP_3DP. In addition, because of technical feasibility and printing costs, we only included the spine body but not peripheral bones, which have a slightly higher density compared to muscle. Nevertheless, the dosimetric characteristics of the PSDP_3DP were matched well with those of the patient, with a dose difference within 5%. Thus, the different densities of materials appeared to compensate for each other.

The PSDP_3DP was designed to physically imitate a real patient using three different types of material densities representing muscle, bone, and titanium fixation hardware. While it would be ideal to print a PSDP_3DP using multiple materials at the same time, our model was assembled from 20 separately printed pieces generated using three different types of 3DP for several reasons. Although some manufacturers have developed multi-material 3DP, their utility is limited in terms of color and material options. On the other hand, a 3DP technology capable of printing structures with heterogeneous and controllable densities has not yet been developed, especially in terms of combining metallic and nonmetallic components. One problem of such a technology is that the heat produced during metal printing can alter adjacent nonmetal components and reduce adhesion between the two materials.

The PSDP_3DP was designed to allow for dose measurement using film and glass dosimeters; however, these were not used in this study, as our primary focus was to evaluate the accuracy of the manufacturing process. The measurement data also contained errors from the method of dose delivery and dosimetric device itself. Thus, further dosimetric verification may be required before PSDP_3DP can be considered suitable for clinical use.

Our results suggest that PSDP_3DPs can be useful not only for standard DQA, but also special dosimetry applications in the clinic. Specifically, cancer patients who have high-density materials in their body around the target volume frequently receive RT^15^,^26^. Furthermore, clinical implementation of new technologies such as new dose calculation algorithms for TPS or dose derivation techniques for machines requires rigorous testing based on accurate dosimetric evaluation in order to ensure patient safety. In this respect, our study is unique in that, for the first time, a PSDP_3DP was developed for an SBRT patient with MFS based on the patient treatment plan. Importantly, when we directly compared the patient dose distribution with that determined from the dosimetric phantom, we found that the difference between the two was within 5%. Thus, we strongly believe that PSDP_3DP will contribute to enhanced dosimetric accuracy of RT.

The physical accuracy and dosimetric characteristics of PSDP_3DP were comparable to those of a real patient. We noted several technical issues related to 3DP technology for manufacturing the PSDP_3DP in this study. These issues revolved primarily around 3D printing techniques and materials, but, these are expected to be resolved in the near future, especially given the rapid advances currently being made in 3DP technology. Given such advances, we anticipate development of a new environment for verifying radiation dose using PSDP_3DPs, in which they are generated directly in the planning room in order to confirm the treatment plan.

## Methods

### Patient selection and contouring of the 3D printed-structure

This dosimetric study did not involve any human or animal experiments. A retrospective study of one patient who underwent SBRT for recurrent thoracic spine metastasis using Novalis Tx^TM^ (Varian Medical Systems, USA) was conducted as part of a protocol approved by the Samsung Medical Center Institutional Review Board (SMC IRB, 2012-08-088-002). All data collection and analysis were conducted in accordance with the institutional review board. The patient had previously undergone surgical decompression with MFS. Contrast-enhanced computed tomography (CT) for RT simulation was performed using a 1.25 mm slice thickness with the patient in the supine position. CT image was transferred to the TPS (Pinnacle^3^, Version 9.10, Philips, USA) using the DICOMRT protocol. Gross tumor volume (GTV) and clinical target volume (CTV) were determined for SBRT planning. The OARs included the spinal cord, lungs, and esophagus.

An intensity-modulated radiation therapy (IMRT) plan for treatment consisting of 11 fixed-field step-and-shoot beams was generated with the direct machine parameter optimization module, which was provided by the TPS manufacturer for dose optimization and conformed to RTOG 0631 dose constraint guidelines^1^. The plan was calculated using the collapsed-cone convolution algorithm on a 2-mm dose grid for a 16-Gy prescription dose with single fraction to the GTV, while 10-Gy was prescribed for the CTV.

**Figure 4:**
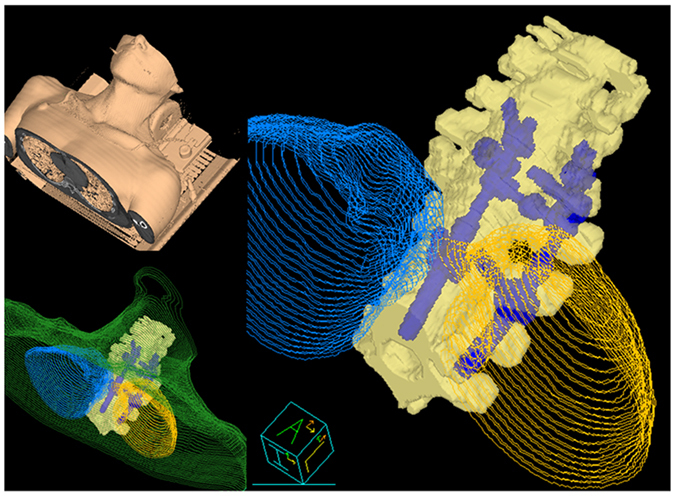
Contours of the external body (green), spines (yellow), and metallic fixation screws (blue, MFS) were delineated using CT images of a patient with MFS who underwent spine to design a patient-specific dosimetric phantom. The ribs and lungs were not included in the three-dimensional printed.

For the purpose of 3DP, the external body contour, spine, and MFS of the patient were also delineated (Fig. 4). The ribs and lungs were not included in the 3D printed structure due to issues of technical feasibility and printing costs. Instead, the medium representing the internal organs was planned such that it had the same density as muscle.

### Radiation-aided design (RAD) procedure for PSDP_3DP

**Figure 5:**
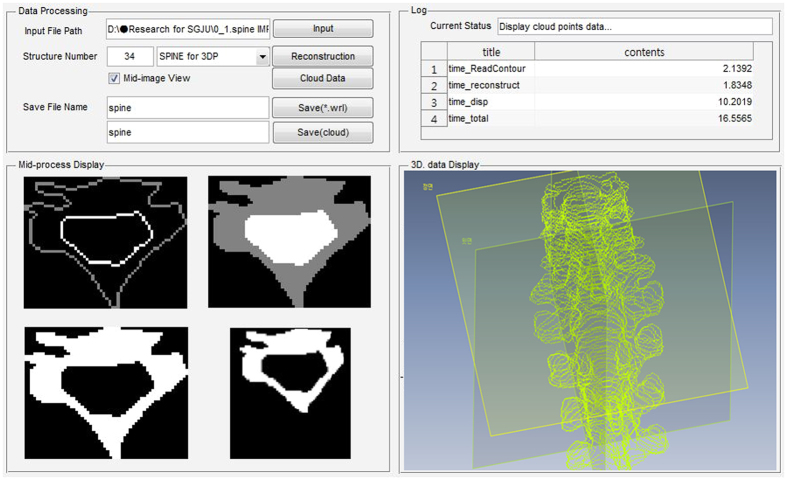
The in-house software program has two main functions, including contour data conversion from DICOMRT to 3D structure set for 3DP and simple 3D structure design that uses Matlab (Mathworks, USA).

To facilitate PSDP_3DP, we developed RAD, which has two main functions. Specifically, RAD converts contour data from DICOMRT to a 3D structure data set for 3DP and design a PSDP_3DP (Fig. 5). After confirming the treatment plan, we selected the point of interest (POI) for DQA with a glass dosimeter for absolute dose and dosimetric film for relative dose verification. In order to insert these dosimeters in the PSDP_3DP for future use, we delineated the outer shape of the glass dosimeter and marked a cut face for film insertion at the POI. All of the contour data was exported to RAD using the DICOMRT file format, which contains contour data with geometrical coordinates based on the beam isocenter. The first step in RAD was to import and reconstruct the data in cloud data format in a 3D virtual space. Next, the cloud data representing contours was converted into an STL (STereoLithography, 3D Systems, Inc., USA) file format^27^.

**Figure 6:**
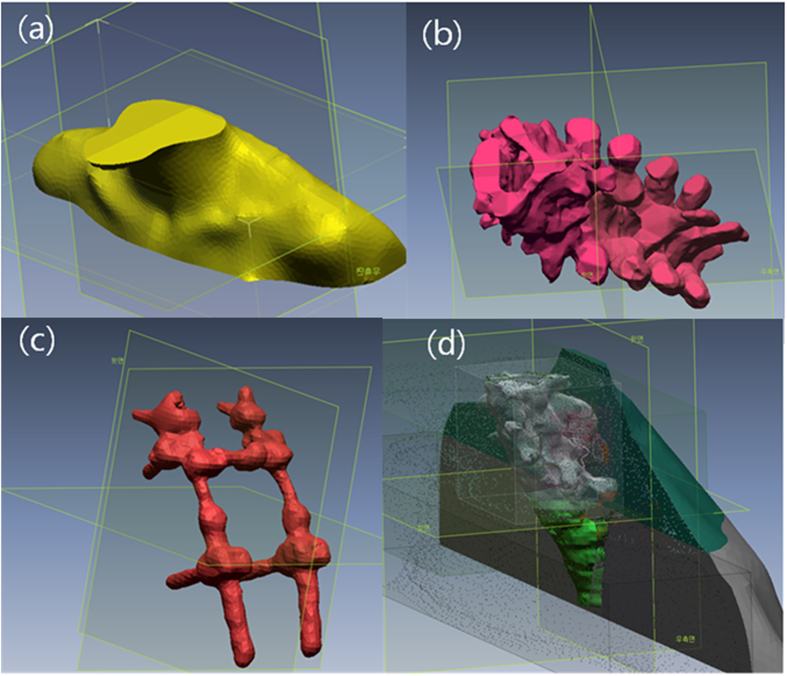
A mesh fitting process was employed to obtain a smooth surface (**a**–**c**). Due to the technical feasibility of assembling OARs by 3D printing, the PSDP_3DP was designed such that it had a hollow structure that could be filled with a muscle-equivalent liquid. All structures were combined into a virtual PSDP_3DP in the same coordinate system.

We performed a mesh fitting process to obtain a smooth surface (Geomagic Design X, 3Dsystems, USA) (Fig. 6a–c)^28^. Because of the technical feasibility of assembling OARs after 3D printing, the PSDP_3DP was designed such that it had a hollow structure that could be filled with a muscle-equivalent liquid. Specifically, an external body shell was generated by applying a 5 mm inner wall offset (5 mm thickness) to the external body surface. All structures were combined into the virtual PSDP_3DP based on the same coordinate system. We removed duplicate regions (overlap with other organs) from the external body, followed by spine body, MFS, and glass dosimeter insertion hole based on patient anatomy through Boolean subtraction operation on polygons (https://en.wikipedia.org/wiki/Boolean_operations_on_polygons) (Fig. 6d). The virtual model of the PSDP_3DP was cut into four sections to allow for insertion of film in the axial and coronal planes. Two holes for each section were generated on the external body shell to inject the liquid-filling material, which was an agar liquid meant to represent muscle. The final PSDP_3DP design consisted of 20 different pieces of three different materials with densities conforming to muscle, bone, and titanium for MFS.

### 3D printing and assembly of PSDP_3DP

The virtual PSDP_3DP was realized by first printing the 20 pieces using three different 3DP techniques with compatible materials (Fig. 1a). First, the external body shell pieces were printed using a multi-jet 3DP (Projet^®^5000, 3Dsystems^®^, USA), which uses a UV- curable acrylic plastic (UVAP) printing material. Parts of the spine body were printed using a Powder Bed and inkjet head 3DP (PBP, Z printer, 3Dsystems^®^, USA), which uses a plastic powder material. In order to imitate the clinical situation, titanium (Ti) metallic screws, which are commonly used in the clinic, were printed by direct metal laser sintering (DMLS, Mlb cusing R, Concept Laser GmbH, Germany). A post-cleaning procedure was performed for all of the pieces of the PSDP_3DP.

We assembled the 20 pieces of the PSDP_3DP, beginning with the spine body and metallic screws and finally the external body shell (Fig. 1b). For convenience of the assembly process, holes on the spine body were slightly enlarged through a boring process to allow the metal fixation screws to be easily inserted. Finally, the external body shell was filled with an agar liquid through holes on the external body shell, which were closed using plugs manufactured with the same material as the external shell (Fig. 1c and d).

### Evaluation of the geometric and dosimetric accuracy of PSDP_3DP

To evaluate the geometric accuracy of the PSDP_3DP, CT images of the PSCD-3DP were acquired with a 1.25 mm scan thickness (Fig. 1d) and compared with that of the patient CT. The CT image of the PSDP_3DP were transferred to the TPS using the DICOMRT protocol. The CT image of the PSDP_3DP was registered to the patient CT image using the rigid body registration algorithm provided by the TPS. The contours of the PSDP_3DP, namely, those of the external body, spine, and MFS, were delineated under the same condition used for the patient by the same oncologist, while PTV, CTV, lung, and spinal cord information was transferred without change. We calculated the volume and mean density of each contour set by applying the Hounsfield unite (HU) to density conversion equation for the CT scanner.

For quantitative analysis of geometric accuracy, we employed the Dice similarity coefficient (DSC), which is defined as twice the overlap of A and A′, divided by the sum of the volume of A and A′^29^:


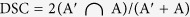


where A is the original patient contour delineated from the patient CT image, and A′ is the same contour delineated on the CT image of the PSDP_3DP. If A′ and A have no overlap, then the DSC is 0, while the value of DSC approaches 1 as the contours become identical.

We next calculated the radiation dose to verify the dosimetric accuracy of the PSDP_3DP. The same beam parameters optimized for the real patient treatment were imported into the registered CT image set of the PSDP_3DP based on the same isocenter position with the DICOMRT protocol using the adaptive plan function provided by the TPS. A density override for image artifacts was performed for MFS (Ti, ρ = 4.5 g/cm^3^), body (muscle, ρ = 1.05 g/cm^3^), and spine (ρ = 1.5 g/cm^3^) according to the value obtained from the PSDP_3DP, as discussed in the result session. In addition, the density of the lung contour imported from the patient plan was overridden as 0.3 g/cm^3^. Dose distribution was calculated by applying the same dose calculation algorithm and grid resolution without additional optimization. Two treatment plans, one based on the real patient and one from the PSDP_3DP, were exported from Pinnacle to the RayStation TPS (RaySearch, Stockholm, Sweden) for convenience of dosimetric analysis. From these two sets of plans, we compared dosimetric characteristics, including dose volume histogram (DVH) and mean dose for CTV, GTV, spinal cord, and external body contour. In addition, two-dimensional dose difference maps were calculated for the axial plane in the mid-region of the CTV.

## Additional Information

**How to cite this article**: Oh, D. *et al*. Development of patient-specific phantoms for verification of stereotactic body radiation therapy planning in patients with metallic screw fixation. *Sci. Rep.* **7**, 40922; doi: 10.1038/srep40922 (2017).

**Publisher's note:** Springer Nature remains neutral with regard to jurisdictional claims in published maps and institutional affiliations.

## Figures and Tables

**Table 1:** Comparison of geometric and dosimetric characteristics of the PSDP-3DP and patient TPS data.

	Geometric accuracy					Dosimetric characteristics			
	External body		Spine		MFC^*^	Mean dose (cGy)			
	Volume (cm^3^)	Density (g/cm^3^)	Volume (cm^3^)	Density (g/cm^3^)	Volume (cm^3^)	GTV	CTV	Spinal cord	External body
Patient	4438	0.99 ± 0.37	156	1.29 ± 0.34	20	1513	1311	425	285
PSDP_3DP^†^	4256	1.07 ± 0.29	166	1.53 ± 0.46	22	1506	1304	442	277
Difference (%)	−4.1%	7.5%	6.4%	15.5%	10.0%	−0.5%	−0.5%	4.0%	−2.8%
DSC^‡^	0.98	N/A	0.91	N/A	0.89	N/A			

Abbreviations: MFC^*^ = metalic fixation screw; PSDP_3DP^†^ = patient-specific dosimetric phantom; DSC^‡^ = Dice similarity coefficient.
